# Blancard’s Pills of Iodide of Iron

**Published:** 1856-03

**Authors:** 


*Blancard's Pills of Iodide of Iron.* — Five years ago, M. Blancard, a pharmacien of Paris, proposed an unchangeable pill of the iodide of iron, made directly from its elements, which was officially approved by the French Academy of Medicine. The excellence of this preparation was generally acknowledged, and it is already, in France, the most common form for the administration of iodide of iron. Our pharmaceutical authorities at Philadelphia, however, adhere to the saccharine solution which Dr. Jackson introduced many years ago, and Prof. Bache declares that the solid iodide "might well be dispensed with." Practitioners will differ sometimes from the chemists, and so it has proved in this case. It is found that, notwithstanding the assurances of the self-constituted authorities, the syrup solution of iodide of iron *does* undergo change: that it often injures the teeth, disagrees with the stomach, and contains free iodine. Consequently, as ou dispensatory-authors and colleges of pharmacy simply advise us, if we must have a pill, to evaporate their syrup, or to use the antiquated and unreliable process of Callond, practitioners have found it of advantage to import M. Blancard's preparation, which is now very commonly prescribed, not only in New York and Boston, where there are agencies for the sale of it, but in many remote country towns. And here we may take the liberty of recommending to the gentlemen who have taken on themselves the direction of pharmaceutical matters in this country, that they should not be too dictatorial or dogmatic, if they expect to retain the authority which has been conceded to their talents and learning.

With these preliminaries, we give at length the process for preparing Blancard's pills, which we take from the *Bulletin de l' Académie de Medecine.* It is founded on the volatility of ether, and the insolubility of the iodide of iron in this vehicle:

Take of iodine *seventy-seven grains;* Iron filings *thirty-seven grains;* Distilled water *two and a half drachms;* Honey *one drachm and thirty-four grains;* Absorbent powder (say powder of Althæa) *a sufficient quantity.* Make 100 pills.

Place the water, iodine, and iron in a Florence flask; shake the vessel as the reaction takes place; filter the green liquor that results, into a small iron capsule, the weight of which is known. Wash the flask, and filter with two and a half additional drachms of water, slightly sweetened with a portion of the honey to be used in making the pills. Pour both liquids into the capsule, and evaporate, at first rapidly, then at a gentler heat, until the weight of the mixture is equivalent to the combined weight of the iodine and honey (171 grains, or ʒiij. nearly). Add a sufficient quantity of powdered althæa root, or, still better, equal parts of althæa and liquorice powder, about ʒij. Divide the mass into four equal parts; roll each part in powdered iron. Make each mass into a cylinder on an iron slab; divide each cylinder into twenty-five pills, and roll each pill in powdered iron, to cover the iodide exposed by the spatula. Expose the pills to a gentle heat, that they may contract no moisture, and proceed at once to the second part of the process—varnishing the pills.

Make a solution of balsam of Tolu in three parts of ether. Place the pills in a porcelain capsule, pour on them a portion of ethereal tincture, and impress a rapid movement of rotation, that the pills may be moistened on every side, and that the ether may evaporate rapidly. As soon as the pills begin o stick together, throw them on a dry surface, separating those that are agglutinated, and leave them exposed to the air for twenty-four hours; then dry them over a stove at a gentle heat.

It is well to give them a second coating of varnish. Blancard puts them in a bottle with a stopper covered with silver, which is at once tarnished by the vapor of free iodine.

Each pill contains about one grain of iodide of iron, and one fifth of a grain of powdered iron on its surface. Two to four pills daily is the ordinary dose in chlorotic, scrofulous, tuberculous, and syphilitic diseases.—C. E.—*Virginia Med. & Surg. Journal.*

